# Digital Pattern Recognition for the Identification of Various Hypospadias Parameters via an Artificial Neural Network: Protocol for the Development and Validation of a System and Mobile App

**DOI:** 10.2196/42853

**Published:** 2022-11-25

**Authors:** Irfan Wahyudi, Chandra Prasetyo Utomo, Samsuridjal Djauzi, Muhamad Fathurahman, Gerhard Reinaldi Situmorang, Arry Rodjani, Kevin Yonathan, Budi Santoso

**Affiliations:** 1 Department of Urology Faculty of Medicine Universitas Indonesia Jakarta Indonesia; 2 YARSI E-Health Research Center Faculty of Information Technology YARSI University Jakarta Indonesia; 3 Department of Internal Medicine Faculty of Medicine Universitas Indonesia Jakarta Indonesia

**Keywords:** artificial intelligence, digital recognition, hypospadias, machine learning

## Abstract

**Background:**

Hypospadias remains the most prevalent congenital abnormality in boys worldwide. However, the limited infrastructure and number of pediatric urologists capable of diagnosing and managing the condition hinder the management of hypospadias in Indonesia. The use of artificial intelligence and image recognition is thought to be beneficial in improving the management of hypospadias cases in Indonesia.

**Objective:**

We aim to develop and validate a digital pattern recognition system and a mobile app based on an artificial neural network to determine various parameters of hypospadias.

**Methods:**

Hypospadias and normal penis images from an age-matched database will be used to train the artificial neural network. Images of 3 aspects of the penis (ventral, dorsal, and lateral aspects, which include the glans, shaft, and scrotum) will be taken from each participant. The images will be labeled with the following hypospadias parameters: hypospadias status, meatal location, meatal shape, the quality of the urethral plate, glans diameter, and glans shape. The data will be uploaded to train the image recognition model. Intrarater and interrater analyses will be performed, using the test images provided to the algorithm.

**Results:**

Our study is at the protocol development stage. A preliminary study regarding the system’s development and feasibility will start in December 2022. The results of our study are expected to be available by the end of 2023.

**Conclusions:**

A digital pattern recognition system using an artificial neural network will be developed and designed to improve the diagnosis and management of patients with hypospadias, especially those residing in regions with limited infrastructure and health personnel.

**International Registered Report Identifier (IRRID):**

PRR1-10.2196/42853

## Introduction

Hypospadias is the most prevalent congenital anomaly of the penis, with an estimated incidence of 0.4 to 8.2 cases per 1000 live births [1]. However, most of the parents and families of those with hypospadias experience anxiety and uncertainty regarding the information about hypospadias [2]. Moreover, there remains some confusion in the diagnosis of hypospadias even among clinicians [3]. The confusion among both clinicians and parents delays the diagnosis and treatment of hypospadias, exacerbating the symptoms and lowering the quality of life of those affected [2].

Hypospadias is a multidimensional problem that requires a multidisciplinary approach. Due to the possibility of it being one of the symptoms of disorders of sexual development, various types of specialistic care are required for the most optimal outcome [4]. However, there are shortages of multidisciplinary teams that are capable of performing the diagnosis and surgery needed, especially in low-income countries such as Indonesia [4].

The diagnosis of hypospadias is usually based on clinical observation. However, the accurate diagnosis of hypospadias may prove to be difficult, as its phenotypes widely vary. Different diagnoses for the same phenotypes may result in variable surgical outcomes, even among experienced surgeons who perform the same procedure [5]. Although a few scoring systems exist for standardizing the clinical diagnosis of hypospadias, the lack of complete agreement among clinicians remains an issue [6,7]. There are a few established scoring systems for diagnosing the severity of hypospadias, such as the Glans, Meatus, Shaft (GMS) score; Meatus, Chordee, Glans, Urethral Plate Quality (MCGU) score; and Hypospadias Objective Penile Evaluation score [6,8,9]. Merriman et al [7] showed that complete agreement for a scoring system among clinicians could only be achieved in 78%-85% of their cases. Therefore, some studies utilized photos and videos to help diagnose hypospadias [10,11].

Various studies have been done to address this problem. One such study used standardized photographs, which were taken by using a professional camera, for measurements of various parameters of hypospadias [12]. Another used digital photographs taken by clinicians for media communication in hypospadias care [13], and one study even utilized parents’ video cameras for postoperative follow-up examinations [14]. However, there has been no study utilizing artificial intelligence, image recognition, and parents’ mobile cameras to help diagnose hypospadias.

This protocol was designed to develop a system and a mobile app with artificial intelligence and image recognition capabilities, which are thought to be beneficial in improving the diagnosis and management of hypospadias cases, especially in low-income countries such as Indonesia.

## Methods

### Study Design

Ours is an observational study that will use a prospective cohort design for the development of a digital pattern recognition system for the identification of various hypospadias parameters via an artificial neural network (ANN). The digital pattern recognition system will be applied to digital images that are taken by the parents or guardians. Afterward, the system will be applied to a mobile app. The population for our study will be children with suspected hypospadias who are admitted or referred to hospitals in Indonesia.

### Ethics Approval

The approval for the protocol of our study was granted by the Medical Research Ethics Committee, Universitas Indonesia, in April 2022 (ethical clearance number: KET-413/UN2.F1/ETIK/PPM.00.02/2022).

### Consent to Participate and Consent for Publication

The consent for the capture and publication of the images needed in the study will be obtained from the parents of patients with hypospadias as a part of standard care. The images will be used as a clinical reference. Consent will be requested after the parents and their children receive an explanation from the researcher.

The photographs for the study will be taken by the parents or guardians of the participants, using their mobile cameras. The photographs will then be securely uploaded via the internet to our encrypted database, without any identifiers. Photographs with an identifier, such as a face, will be marked as invalid and will be deleted from our database. The photographs will only be seen by the parents and the researcher. The photographs chosen as examples for the publication will also be randomized according to the parameters needed for the publication.

This protocol was prepared according to the SPIRIT (Standard Protocol Items: Recommendations for Interventional Trials) 2013 checklist for reporting a protocol study [15].

### Eligibility Criteria and Recruitment Procedures

The inclusion criteria for participants in the hypospadias (case) group are as follows: children aged <18 years and (2) children with suspected hypospadias. Those for the control group are (1) children aged <18 years and (2) children without suspected hypospadias.

The exclusion criteria for the hypospadias (case) group are (1) a history of hypospadias repair and (2) the refusal to participate in the study. The exclusion criterion for the control group is the refusal to participate in the study.

The drop-out criterion is death during the follow-up period.

The participants of our study will be recruited by using the consecutive sampling method. Parents of patients who are eligible to participate as study participants during the study duration will be given an explanation about the study process and be asked whether they want their children to participate in the study.

### Clinical Outcomes: Hypospadias Parameters

The clinical outcomes measured in our study will be expressed as hypospadias parameters. The hypospadias parameters in our study will be defined as diagnostic features that define the severity of hypospadias [6,7]. These parameters are known to define the prognosis of the surgical management of patients [6]. It is expected that the system will be able to recognize these diagnostic features after sufficient training. The hypospadias parameters and their categorizations in the study will be (1) hypospadias status (hypospadias or nonhypospadias), (2) meatal location (glanular, coronal, distal shaft, proximal shaft, or penoscrotal), (3) meatal shape (normal or abnormal), (4) the quality of the urethral plate (intact or divided), (5) glans diameter in millimeters, and (6) glans shape (normal or abnormal).

The clinical outcomes will be measured preoperatively and 1 month postoperatively.

### Model Development, Training, and Testing

Hypospadias and normal penis images from an age-matched database will be used to train the ANN. Images of 3 aspects of the penis (ventral, dorsal, and lateral aspects, which include the glans, shaft, and scrotum) will be taken from each participant. The images will be labeled with the following hypospadias parameters: hypospadias status, meatal location, meatal shape, the quality of the urethral plate, glans diameter, and glans shape. The data will be uploaded to train the ANN.

In our study, a customized ANN based on a deep learning architecture will be used to develop an image recognition model for determining various hypospadias parameters. The software will be trained to recognize various hypospadias parameters via label boxes as a part of the preprocessing phase. The labels will be filled after consensus among 3 pediatric urologists.

Following the training period, the evaluation of the ANN will be done by comparing the results of the ANN and the three pediatric urologists’ evaluations for a single image. Typically, the interrater reliability test for hypospadias is done by comparing scores measured via scoring criteria, such as the GMS score or the MCGU score [6,7]. The results obtained by the ANN will also be converted according to the scoring criteria. The agreement among raters will be assessed by using the intraclass correlation coefficient as the gold standard.

**Figure 1:**
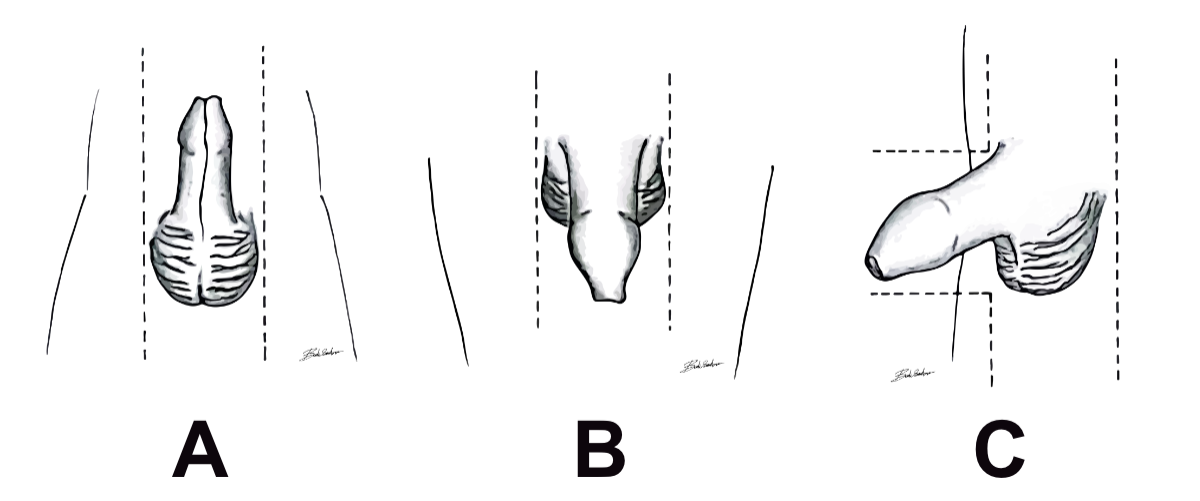
Example drawings that will be presented before a photograph is taken. (A) Ventral view. (B) Dorsal view. (C) Lateral view.

The photographs used for the study will be taken by using the same standard methodology and be of the same quality, using the parents’ or clinicians’ phones (ie, via a mobile app). An example drawing and guiding lines will be presented before a photograph is taken (Figure 1).

### Data Analysis

The sample size was estimated for a prespecified power of 90%, while the α value was set at <.05. The clinical characteristics of the participants and all hypospadias parameters will be presented descriptively. Intrarater and interrater analyses among pediatric urologists will be performed, using the Fleiss κ statistical analysis. The κ score between the ANN results and pediatric urologists’ examination results will be calculated by using SPSS for Macintosh, version 25.0 (IBM Corporation). The data will be deemed statistically significant if the *P* value is <.05. In addition, accuracy, precision, recall, and *F*_1_ score values will be computed to measure the performance of the recognition model.

## Results

The development of the system and a mobile app started in September 2022, and the recruitment of participants is planned to start in December 2022. The study results are expected to be available by the end of 2023.

## Discussion

It is expected that after being trained on images of patients with hypospadias and age-matched controls, the image recognition model will be able to differentiate between a normal penis and a hypospadias penis, determine meatal location and shape, and measure glans size and quality. The parameters identified by the model will prove to be useful in determining the prognosis of patients with hypospadias.

The diagnosis of hypospadias remains a challenge even among clinicians worldwide [1]. Even among experts, there are many debatable aspects of hypospadias, such as the diagnosis method, the assessment of severity, the classification of hypospadias, and hypospadias treatment [16].

There are some studies that have already used a similar approach to that of our study. Previously, Han et al [10] studied the validity and reliability of guardian-conducted voiding videos for postoperative evaluation following hypospadias surgery. In their study, it was shown that the videos taken by the guardians were acceptable and beneficial for home-based postoperative assessment. However, the videos taken during the assessment were compared to web-based observations by pediatric urologists. Therefore, the applicability of the test was limited by the number of pediatric urologists available and time constraints.

Fernandez et al [11] studied the application of digital pattern recognition and artificial intelligence for the classification of hypospadias. After being trained with 1169 images of hypospadias cases, their model was reported to have about 90% accuracy, surpassing the interrater analysis results among expert pediatric urologists. However, the hypospadias images were taken by the clinicians before surgery, limiting the applicability of the model.

Herein, we present a protocol for the assessment of hypospadias by using digital pattern recognition for the identification of various hypospadias parameters. Using a customized ANN with a deep learning architecture, we will train an accurate model with data from a limited number of participants. We hope that the model will facilitate the diagnosis of hypospadias and ease the burden of hypospadias management among clinicians. However, a previous study showed that there are challenges in encouraging clinicians to use mobile apps in the clinical setting, such as convincing clinicians that an app will be useful [17]. Therefore, we chose to include the parents and guardians of patients with hypospadias due to the limited number of pediatric urologists in low-income countries—an approach that was previously validated by Türk et al [14] for postoperative follow-up assessments among children with hypospadias.

The use of mobile health apps among adults has been previously studied, and it has been deemed a feasible option for dealing with various health issues in Indonesia. Agustina et al [18] previously developed a mobile app for obesity management in Indonesia, especially in urban areas. Even in rural areas, the use of mobile apps has proven to be beneficial for the follow-up assessment of patients [19]. We also hope that our app will be beneficial even in the rural areas of low-income countries.

One of the limitations of our study is the sheer number of images needed to train the model. Based on a previous study, more than 900 images of patients with hypospadias are required to have a system with over 90% accuracy [11]. However, there are only a limited number of health centers with a sufficient number of patients with hypospadias. Therefore, breakthroughs in ANN development and extreme learning techniques are necessary for health centers with a limited number of patients with hypospadias.

Another limitation of our study is the unpredictability of the digital images, which will be taken by the parents or guardians of the patients. Due to the variability in digital camera quality among mobile phones, the quality of the digital images may vary, creating another hurdle for the digital pattern recognition system.

With the advancement of information and communication technology, the advancement of health information technology is expected. Hypospadias, as a prevalent congenital abnormality with recognizable anatomical features, may serve as an example for the use of artificial intelligence in health.

## Appendix

Multimedia Appendix 1 Peer-review reports from the Kementrian Riset Dan Teknologi / Badan Riset Dan Inovasi Nasional Deputi Bidang Penguatan Riset Dan Pengembangan (Ministry of Research and Technology / National Research and Innovation Agency - Deputy for Strengthening Research and Development) (Jakarta, Indonesia).

## Abbreviations

ANN: artificial neural network
GMS: Glans, Meatus, Shaft
MCGU: Meatus, Chordee, Glans, Urethral Plate Quality
SPIRIT: Standard Protocol Items: Recommendations for Interventional Trials

## Data Availability

The data sets that will be generated and analyzed during the study will be available from the corresponding author on reasonable request.
