# Supplementary material for: Digital Pattern Recognition for the Identification of Various Hypospadias Parameters via an Artificial Neural Network: Protocol for the Development and Validation of a System and Mobile App
Source: JMIR Res Protoc. 2022 Nov 25;11(11):e42853. doi: 10.2196/42853 (PMC9736751; doi:10.2196/42853)
Supplement: Multimedia Appendix 1 [file resprot_v11i11e42853_app1.pdf]

KEMENTRIAN RISET DAN TEKNOLOGI /
BADAN RISET DAN INOVASI NASIONAL
DEPUTI BIDANG PENGUATAN RISET DAN PENGEMBANGAN
Gedung BJ Habibie Lantai 19 – 20, Jalan M.H. Thamrin Nomor 8, Jakarta 10340
Telepon: (021) 3169707; Faksimile: (021) 3101728, 3102368
Laman: www.risbang.ristekbrin.go.id

Reviewers' Comments for Author's Rebuttal

Manuscript titled "The Digital Pattern Recognition for Identification of Various Hypospadias Parameters using Artificial Neural Network: a Research Protocol"

Please write down your response or revision in **"Author's Response / Revision"** column. Locate the changes you've made or the sentence that you refer to as their page and line in **"Location in Text"** column. Please also mark the changes you have made **in your manuscript** by highlighting or changing the font color.

Reviewer

No.	Aspect	Comments	Author's Response / Revision	Location in Text
	Title	**REVIEWER 1**<br>Appropriate<br><br>**REVIEWER 2**<br>The use of artificial neural network is an important part in this study. Thus, it should be added to the title.	The title has been changed accordingly.	Title

	Abstract	**REVIEWER 1**<br>1. Please specify the image aspects taken from the patients for this study.<br>2. Please specify all of the parameters measured in the study<br>**REVIEWER 2**<br>1. There are some grammatical errors and mistyping in the abstract	**REVIEWER 1:**<br>1. There are three image aspects taken for the study. Added to the abstract.<br>2. The parameters measured were added to the abstract.<br>**REVIEWER 2:**<br>1. The abstract was revised accordingly.	Abstract<br>Page 2
	Introduction	**REVIEWER 1**<br>1. It think that the introduction needs to explain more about the scope of the problem in Indonesia<br>**REVIEWER 2**<br>-	**REVIEWER 1:**<br>1. Added a section about the lack of multidisciplinary team capable of performing the diagnosis and management of hypospadias and DSD in Indonesia.	Introduction<br>Page 3.
	Methods	**REVIEWER 1**<br>1. As most of the patients would be children, who would provide the informed consent for the patient?<br>2. The clinical outcomes need to be defined as either categorical or numerical data	**REVIEWER 1**<br>1. The informed consent would be provided by the parents or the guardians. Added in the method section.<br>2. Added.	Methods

		**REVIEWER 2**<br>1. The artificial intelligence used in the study should be explained more.<br>2. Who will take the picture of the patient? Is it the clinicians or the parents?	**REVIEWER 2**<br>1. The explanation about AI used in the study was added.<br>2. The picture will be taken by either the parent or the guardian, while the clinicians (pediatric urologists) would provide the label for machine training.	
	Results	**REVIEWER 1**<br>-<br><br>**REVIEWER 2**<br>-	-	Results
	Discussion	**REVIEWER 1**<br>1. Is there any previous study about the use of mobile photography for hypospadias case examination?<br>2. There was a study performed by Pippi Salle et al. with similar methodology. It should be cited in the text.<br><br>**REVIEWER 2**<br>1. The limitation of the protocol needs to be explained	**REVIEWER 1**<br>1. There has been some studies about the use of digital photography for follow-up examination of hypospadias cases. However, the use of both mobile application, digital photography, and AI has not been performed yet.<br>2. Cited and explained in the discussion section.<br>**REVIEWER 2**<br>1. The limitations of the study were added	Discussion
